# First case of T-cell/histiocyte-rich-large B-cell lymphoma presenting with duodenal obstruction

**DOI:** 10.3402/ljm.v8i0.22955

**Published:** 2013-12-16

**Authors:** Ali Riza Köksal, Huseyin Alkim, Meltem Ergun, Salih Boga, Mehmet Bayram, Canan Alkim, Ozlem Ton Eryilmaz

**Affiliations:** 1 Department of Gastroenterology, Sisli Etfal Education and Research Hospital, Istanbul, Turkey; 2 Department of Pathology, Sisli Etfal Education and Research Hospital, Istanbul, Turkey

Gastrointestinal tract is the most common site of extranodal non-Hodgkin's lymphoma. Small bowel involvement accounts for 20–30% of all gastrointestinal lymphomas (1). Among all intestinal lymphomas, the ileum is the most common involved area, with duodenal involvement only accounting for a small proportion (2). The most frequent histological type is diffuse large B-cell lymphoma (DLBCL). T-cell/histiocyte-rich-large B-cell lymphoma (TCHRBCL) is an uncommon morphologic variant of B-cell lymphoma (3, 4). Duodenal involvement of TCHRBCL has not been reported previously. We hereby report this first case of TCHRBCL of the duodenum.

## Case report

**Fig. 1:**
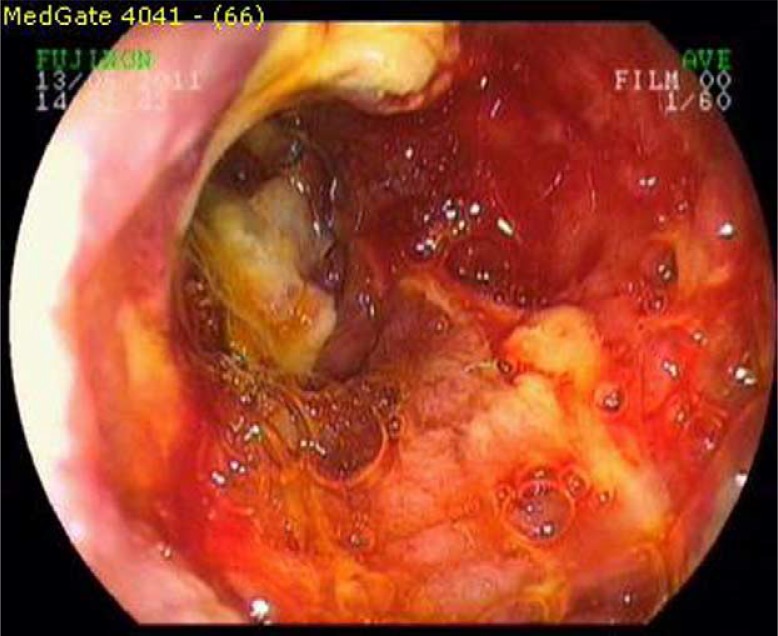
Lesion at the third part of the duodenum (before treatment).

A 28-year-old man was admitted to our clinic with nausea, vomiting, abdominal pain, and weight loss (10 kg), which he had experienced for 4 months. His medical history was unremarkable. Physical examination showed mild tenderness at the epigastric region. Laboratory tests were normal except for mild iron-deficient anemia (hemoglobin: 11.6 g/dl; iron: 19 µg/dl; iron binding capacity: 272 µg/dl). Gastroscopic examination showed an ulceroinfiltrating mass lesion with a fragile surface and deep exudations at the third part of the duodenum (Fig. 1).

**Fig. 2:**
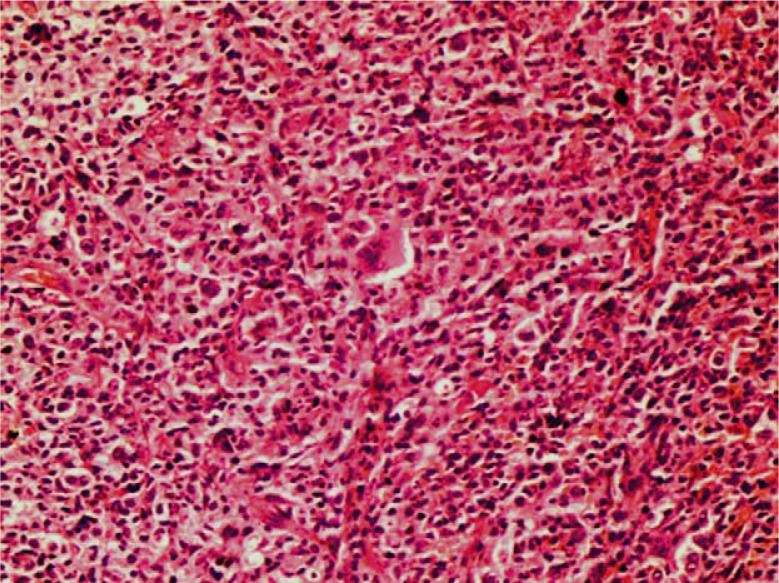
Rare large neoplastic cells with large nuclei and prominent nucleoli between small lymphocytes and histiocytes.

**Fig. 3:**
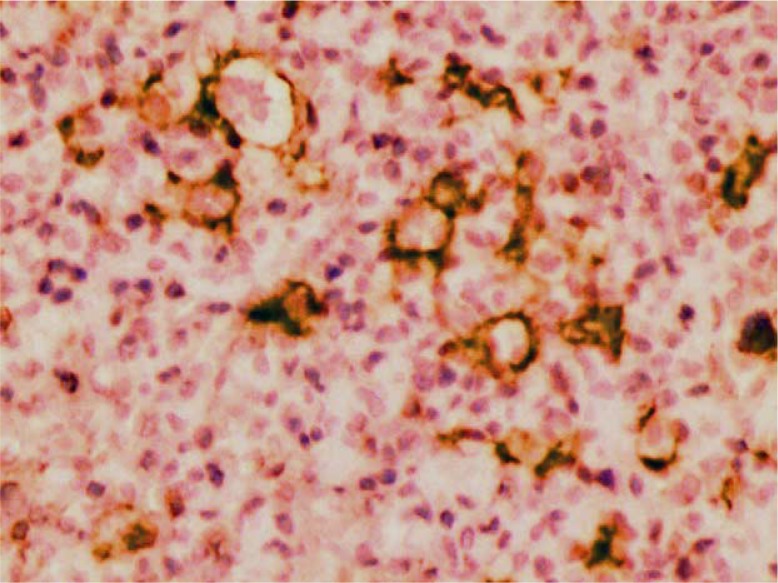
Neoplastic cells showing membranous staining with CD 20.

Histopathologic examination of the biopsies showed small lymphocytes and histiocytes in the lamina propria with atypical morphology, together with large malignant cells having pleomorphic vesicular nuclei and prominent nucleoli (Fig. 2). Hodgkin's lymphoma, anaplastic large B-cell lymphoma, T-cell lymphoma, malignant epithelial tumors, and germ cell tumors were included in the differential diagnosis. Immunohistochemical examination of neoplastic cells is as follow: CD20 (+), CD30 (+), CD3 (−), cytokeratin (−), PLAP (−), CD15 (−), EBV (−), LCA (+), LSP1 (+), and fascin (−) (Fig. 3). These findings were reported as TCHRBCL.

**Fig. 4:**
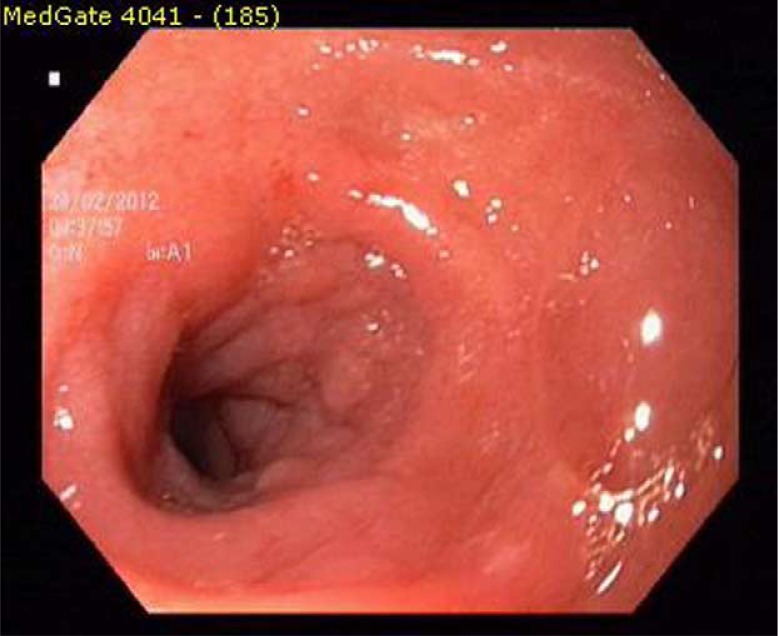
The endoscopic appearance after treatment.

Laboratory examinations showed beta-2-microglobulin: 2697 ng/ml, LDH: 166 U/L. Liver, renal, and thyroid profiles, as well as erythrocyte sedimentation rate, were all normal. Chest radiography was normal. A computed tomography (CT) scan of the abdomen showed an irregular concentric thickened duodenum. There was no abdominal lymphadenopathy or splenomegaly. PET CT was performed for staging, and intense FDG uptake was detected only at the third part of duodenum. Staging was consistent with I E, because there was involvement of only one extranodal place. He was treated with R-CHOP (rituximab, Adriamycin, cyclophosphamide, Oncovin, and prednisolone). The patient had significant improvement in clinical and endoscopic findings at the end of fourth cycle. Chemotherapy was completed to six cycles. A complete cure of the lesion was observed endoscopically and confirmed histopathologically at the end of the therapy (Fig. 4). Both PET CTs done at the end of the treatment and at the second year were normal.

## Discussion

Gastrointestinal tract is the most common site of extra-nodal malignant lymphoma, accounting for 10–15% of all non-Hodgkin's lymphomas. Gastric lymphoma is the most common type (55–70%); while the small intestinal and colorectal involvement occurs in 20–35% and 5–10% of cases, respectively (1, 2). Wang et al. have reported81 cases of primary intestinal lymphoma, with duodenal localization being very rare (2.5%) (5). In addition, the cases with B-cell lymphoma were mostly seen in the terminal ileum and ileocecal valve region.

TCHRBCL is an uncommon variant of large B-cell lymphoma (3, 4, 6). TCHRBCL represents 1–3% of all DLBCL in recent series (5, 7, 8). TCHRBCL cases mostly present in advanced stages ranging from 53 to 91%. In the study of Bouabdallah et al. (9), 50 TCHRBCL cases were found amongst 4500 NHL patients. Gastrointestinal tract involvement was not present. These patients were compared with 150 DLBCL patients. While complete remission was found lower in TCHRBCL group (58% vs. 77%), the 5-year overall survival was not different between the two groups (58% in both groups). While frequency of splenic involvement ranged from 21 to 60%, frequency of liver involvement and that of bone marrow involvement ranged from 4 to 40% (7–10). To date, no case has been reported with duodenal involvement.

Primary gastrointestinal lymphomas are classified as nodular, infiltrative, and ulcerative subtypes according to endoscopic appearance. Although primary lymphomas of the gastrointestinal tract are observed as thickening of mucosal folds, atypical ulcers, erosive, polypoid mushroom-like multiple lesions, and mucosal irregularities endoscopically, they can also be seen as an ulceroinfiltrating mass lesion, like in our case described above (11).

The patient's symptoms included nausea, vomiting, and severe weight loss. Vague intermittent abdominal pain associated with vomiting was probably due to partial intestinal obstruction; therefore, clinical findings of complete obstruction did not exist. There are a few reports that DLBCL presented with intestinal obstruction or perforation; some cases are treated with surgery and some need chemotherapy (12, 13). Fortunately, our case was admitted to hospital at partial obstruction stage and diagnosed with endoscopic biopsies.

In our case, besides histopathologic appearance, malignant epithelial tumors were excluded by cytokeratin negativity, and germ cell tumors were excluded by PLAP negativity in immunohistochemical examination. B-cell origin was demonstrated by CD 20 and LCA positivity. Classical type Hodgkin's lymphoma was excluded by CD15 and Fascin negativity, and TCHRBCL was diagnosed with evaluation of the histological findings and LSP1 positivity (7–10).

CHOP chemotherapy in combination with rituximab is used in TCHRBCL treatment as it is used in all CD20 (+)nodal and extranodal lymphomas (3). Response to the treatment and prognosis of cases with TCHRBCL are similar to the DLBCL cases at the same stage (4, 6, 14). The adjusted International Prognostic Index (IPI) was calculated using the age, LDH, stage, extranodal involvement, and performance score, and the prognosis was determined as‘very good’ (15). Complete remission was achieved with the standard R-CHOP chemotherapy.

Although the gastrointestinal tract is the most common site of extranodal lymphomas, presentation of cases, endoscopic views, involvement sites, and histological types could be variable. High index of suspicion is needed to make a correct diagnosis and complete remission can be achieved by optimal chemotheraphy.

*Ali Riza Köksal* Department of Gastroenterology Sisli Etfal Education and Research Hospital Istanbul, Turkey Email: arkoksal@gmail.com *Huseyin Alkim* Department of Gastroenterology Sisli Etfal Education and Research Hospital Istanbul, Turkey *Meltem Ergun* Department of Gastroenterology Sisli Etfal Education and Research Hospital Istanbul, Turkey *Salih Boga* Department of Gastroenterology Sisli Etfal Education and Research Hospital Istanbul, Turkey *Mehmet Bayram* Department of Gastroenterology Sisli Etfal Education and Research Hospital Istanbul, Turkey *Canan Alkim* Department of Gastroenterology Sisli Etfal Education and Research Hospital Istanbul, Turkey *Ozlem Ton Eryilmaz* Department of Pathology Sisli Etfal Education and Research Hospital Istanbul, Turkey
